# Measurement of ^18^F-FDG PET tumor heterogeneity improves early assessment of response to bevacizumab compared with the standard size and uptake metrics in a colorectal cancer model

**DOI:** 10.1097/MNM.0000000000000992

**Published:** 2019-03-18

**Authors:** Usman Bashir, Amanda Weeks, Jayant S. Goda, Muhammad Siddique, Vicky Goh, Gary J. Cook

**Affiliations:** a Department of Radiology, Barts and London NHS Trust; b Department of Cancer Imaging, School of Biomedical Engineering and Imaging Sciences; c PET Imaging Centre and the Division of Imaging Sciences and Biomedical Engineering, King’s College London; d Department of Radiology, Guy’s Hospital, London, UK

**Keywords:** colorectal cancer, ^18^F-FDG PET, texture analysis, tumor heterogeneity, xenograft

## Abstract

**Purpose:**

Treatment of metastatic colorectal cancer frequently includes antiangiogenic agents such as bevacizumab. Size measurements are inadequate to assess treatment response to these agents, and newer response assessment criteria are needed. We aimed to evaluate ^18^F-FDG PET-derived texture parameters in a preclinical colorectal cancer model as alternative metrics of response to treatment with bevacizumab.

**Materials and methods:**

Fourteen CD1 athymic mice injected in the flank with 5×106 LS174T cells (human colorectal carcinoma) were either untreated controls (*n*=7) or bevacizumab treated (*n*=7). After 2 weeks, mice underwent ^18^F-FDG PET/CT. Calliper-measured tumor growth (Δ_vol_) and final tumor volume (Vol_cal_), ^18^F-FDG PET metabolically active volume (Vol_met_), mean metabolism (Met_mean_), and maximum metabolism (Met_max_) were measured. Twenty-four texture features were compared between treated and untreated mice. Immunohistochemical mean tumor vascular density was estimated by anti-CD-34 staining after tumor resection.

**Results:**

Treated mice had significantly lower tumor vascular density (*P*=0.032), confirming the antiangiogenic therapeutic effect of bevacizumab. None of the conventional measures were different between the two groups: Δ_vol_ (*P*=0.9), Vol_cal_ (*P*=0.7), Vol_met_ (*P*=0.28), Met_max_ (*P*=0.7), or Met_mean_ (*P*=0.32). One texture parameter, GLSZM-SZV (visually indicating that the ^18^F-FDG PET images of treated mice comprise uniformly sized clusters of different activity) had significantly different means between the two groups of mice (*P*=0.001).

**Conclusion:**

^18^F-FDG PET derived texture parameters, particularly GLSZM-SZV, may be valid biomarkers of tumor response to treatment with bevacizumab, before change in volume.

## Introduction

Early detection of response to treatment is an increasingly important goal in personalized medicine. Bevacizumab is a monoclonal antibody to the vascular endothelial growth factor-A (VEGF-A) receptor 1. It selectively targets immature new vessels within tumors and has a vascular maturation effect, whereby highly permeable, immature vessels are replaced by more mature vessels 1–3. These changes manifest as decreasing vascular permeability in tumor regions rich in VEGF-A expression 4,5. Because of the reported survival benefits of bevacizumab, several regimens combining bevacizumab with cytoreductive agents are being used in the treatment of colorectal cancer 1. Like most anticancer therapeutic regimens, early response assessment is critical to identify nonresponders and switch regimens.

Because bevacizumab is not cytoreductive, responding tumors may not decrease in size early during treatment making serial size measurement on morphologic imaging unreliable for response assessment 4. Dynamic computed tomography (CT) and MRI have shown promise in directly measuring the antiangiogenic effect of bevacizumab treatment 2,4. However, the relatively complex nature of some of these CT and MRI protocols limits their use to institutions that have the technical expertise 6. Furthermore, as most of these protocols utilize contrast media, patients with poor renal function cannot benefit from them. ^18^F-FDG PET imaging has also been used to determine metabolic changes in tumors undergoing bevacizumab treatment 2,7,8. Conventionally, metrics of tumor metabolism have been based on whole-tumor assessment, for example, mean standardized uptake value (SUV_mean_) and metabolically active tumor volume (Vol_met_), or measurement of single voxel-values, for example, SUV_max_. With regard to their use as early response indicators in bevacizumab treatment, reports are conflicting. Whereas studies using bevacizumab combined with cytotoxic treatment have shown their potential as early response detectors 7–9, several authors have indicated a noncorrespondence between vascular functional and metabolic response in tumors undergoing bevacizumab monotherapy 10.

With growing interest in measuring tumor heterogeneity, investigators have discovered several ^18^F-FDG PET-derived heterogeneity parameters as a potential alternative biomarker of response to treatment in different cancers 11–15. These heterogeneity parameters encode additional spatial information as opposed to whole-tumor derived metrics such as SUV_mean_, SUV_max_, and Vol_met_, which convey no information regarding spatial tumor heterogeneity. Because of the known tumor heterogeneity in VEGF-A expression 16, it is logical to expect differences in metabolic heterogeneity in treated tumors, with regions of high VEGF-A expression responding to bevacizumab differently from those with low VEGF-A expression.

We hypothesized that ^18^F-FDG PET-derived parameters of spatial heterogeneity may be superior in response assessment compared with whole-tumor-based parameters such as morphologic tumor volume (Vol_met_), maximum metabolism (Met_max_), and mean metabolism (Met_mean_). Hence the objective of this exploratory case–control study was to identify potential new ^18^F-FDG PET biomarkers of early response assessment by comparing ^18^F-FDG PET-derived texture parameters with five conventional parameters, that is, morphologic tumor volume (Vol_cal_), change in morphologic tumor volume (Δ_vol_), Vol_met_, Met_max_, and Met_mean_, in colorectal xenograft models treated with bevacizumab.

## Materials and methods

Animal studies were carried out in accordance with UK Research Council’s and Medical Research Charities’ guidelines on responsibility in the use of animals in bioscience research under a UK home office license. Fourteen CD1/nu/nu athymic nude mice (Charles River, Harlow, UK) were inoculated subcutaneously in the right dorsal flank with poorly differentiated colon adenocarcinoma cells (cell line LS174T; 5×10^6^ cells/mouse) 17. The tumors were allowed to grow for 3 weeks until they reached 6–9 mm diameter by caliper measurement. After 3 weeks, baseline tumor volumes (length×width^2^/2 18) were measured with calipers. Seven mice (treatment group) were treated with bevacizumab (Avastin; Genentech, San Francisco, California, USA) at a dose of 5 mg/kg through intraperitoneal injection on alternate weekdays for six doses. The remaining seven mice were left untreated as control group. After 2 weeks of treatment, final volumes (Vol_cal_) were calculated with calipers in all mice to document tumor growth as percent change in tumor volume: Δ_vol_=100×(final volume−baseline volume)/baseline volume.

NanoPET/CT imaging of both groups of mice was performed with a small animal Bioscan NanoPET/CT (Mediso, Budapest, Hungary). For PET scanning, all mice were anesthetized with inhalational isoflurane (induction isoflurane 4% at 0.8–1 l/min followed by isoflurane 2.5% by anesthetic mask at 0.8 l/min) throughout the scanning with respiratory monitoring and mouse bed temperature maintained at 30°C. All mice received 5±0.2 MBq of ^18^F-FDG PET by tail vein followed by PET imaging acquired at 45–60 min. Images were reconstructed with the ordered subset expectation maximization algorithm using NanoPET/CT scanner embedded software using an energy window of 400–600 keV, coincidence relation of 1–3, and isotropic voxel dimensions of 0.6 mm. After decay correction, voxel uptake values were converted to percentage injected dose per gram of tissue (%ID/g) assuming a tissue density of 1 g/cm^3^. The reconstructed images were then transferred in Digital Imaging and Communications in Medicine format to MATLAB R2012b (MathWorks Inc., Natick, Massachusetts, USA) for analysis.

Texture features were analyzed in ^18^F-FDG PET/CT images using an in-house software implemented in MATLAB (Release 2016b; The MathWorks Inc.). The tumors were delineated with the fuzzy locally adaptive Bayesian (FLAB) algorithm as follows: First, a crude bounding volume was manually drawn encompassing the entire tumor and 1–3 mm of adjacent background region 19. This bounding volume was subjected to FLAB which classified all voxels into three classes: tumor, background, and region of partial volume averaging. The FLAB algorithm used to classify the voxels is described in detail by Hatt *et al.* 19. A final volume of interest (VOI) was obtained by discarding voxels classified as background. The VOI was quantized into 64 equally sized bins. One hundred and fourteen computational features (15 geometric, six model-based, 37 first-order, 21 second-order, and 35 higher-order texture features) were derived from each VOI. Geometric features describe tumor morphological features such as volume, diameter, and surface-to-volume ratio. Model-based features, that is, those derived from the fractal dimension of the VOI, describe texture complexity at multiple scales. First-order texture features such as maximum and mean metabolism (i.e. highest activity voxel in the VOI), skewness, and kurtosis are based on statistical histograms and give no information on the spatial distribution of voxels within the image. Second-order features are derived from gray-level co-occurrence matrices and are locoregional, providing information on pair-wise co-occurrence of gray values in a given direction. Higher-order locoregional texture features include gray-level run length, gray-level size zone, and neighborhood gray tone difference matrices. These take larger neighborhoods of similar gray values in different ways in three-dimension 20–22.

Following imaging, all mice were sacrificed and their tumors excised and analyzed as follows: First, pathologic tumor specimens were stained for CD34 antibodies (a marker of vascularity) using mouse monoclonal antibody to CD34 with biotinylated rat anti-mouse immunoglobulins (Vector Laboratories, Burlingame, California, USA) 23. An enhanced biotin/avidin immunoperoxidase system, Vectastain ABC kit (Vector Laboratories) and liquid 3,3′-diaminobenzidine+chromogen (Dako, Ely, Cambridgeshire, UK) were used to detect antibody binding. Stained sections were viewed under a 10x objective on a Leica DMRB light microscope with an automated stage (Leica Microsystems Ltd, Milton Keynes, UK). Images of whole-tumor sections were captured and tiled using a QICAM FAST1394 color camera (QImaging Corporation, Burnaby, Canada) and Surveyor software (Objective Imaging Ltd, Cambridge, UK) 23. Image analysis was performed with the operator (A.W.) blinded to mouse and treatment groups in (ImageJ; NIH, Bethesda, Maryland, USA) to determine percentage area of the tumor section with positive 3,3′-diaminobenzidine staining. Microvessel density (MVD) was determined by manually counting stained vessels in ImageJ. Stained vessels were highlighted by adjusting the color threshold (hue, saturation, and brightness) and the image was overlaid with a 1000×1000 pixel grid to aid counting.

### Statistical analysis

Continuous variables were reported as mean±SD. The Shapiro–Wilk test was used to determine which variables were normally distributed. For normally distributed variables, differences of means were compared between treated and control mice using the Welch-corrected two-sample *t*-test, whereas for non-normally distributed variables, the differences of medians were compared using the Wilcoxon signed-rank test.

Pathological response to bevacizumab treatment was confirmed by comparing mean MVD of treated versus control mice. To determine whether there was any tumor-growth delay in treated versus control mice, Δ_vol_ were compared as the difference of medians between treated and control mice. Likewise, medians of Vol_met_ and Met_mean_ determined from ^18^F-FDG PET imaging were also compared between the treated and control mice to determine the role of conventional ^18^F-FDG PET in differentiating the two groups.

Finally, ^18^F-FDG PET-derived texture variables were compared between treated and untreated mice. The starting variable-set of 114 variables was reduced by excluding highly correlated variables using ‘caret’ package of R version 3.3.1 (The R Foundation, Vienna, Austria), using 0.8 as the cutoff for pair-wise absolute correlation 24. The remaining variables were compared between treated and control mice for difference of means (or medians as appropriate). Bonferroni correction was applied to the *P* values to mitigate false positives arising from multiple testing using a *P* value of 0.002. Differences between the two groups were summarized in a table and illustrated by bar plots. The length of each bar would indicate fold change in a variable with respect to the control group. Fold change was computed using the formula: (value in the treated group−value in the control group)/value in control group. For example, a fold change of 0 would indicate no change, +1 would indicate that the mean value in the treatment group was twice that of control, and −1 would indicate that the mean value for the treatment group was half that of the control group.

## Results

**Fig. 1:**
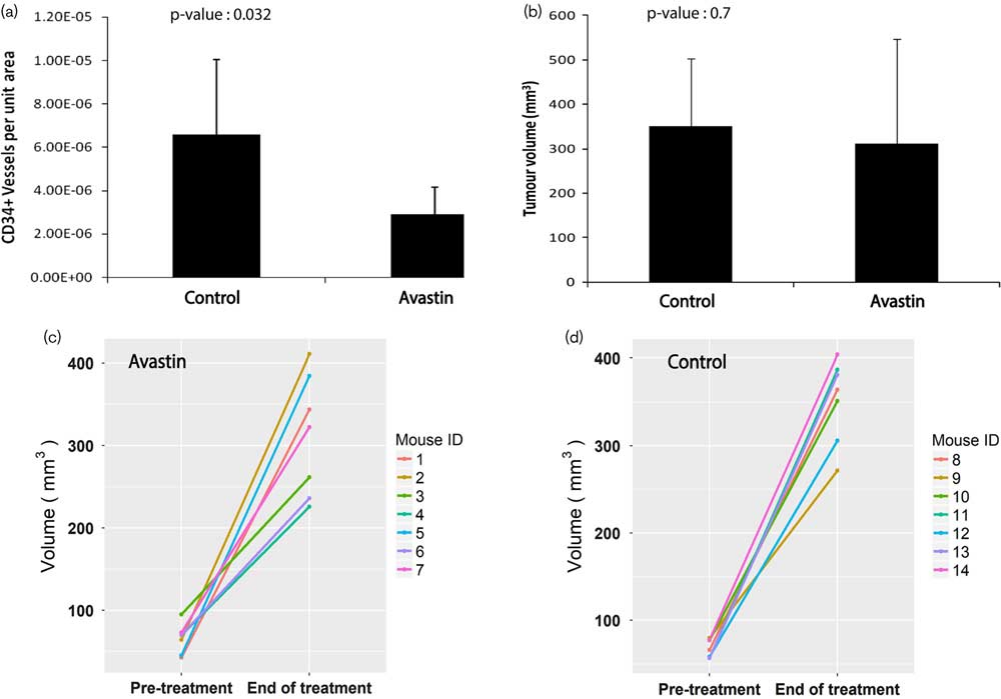
Comparing treated and control mice in terms of differences in MVD (a), volumes (caliper-measured) (b), and temporal change in volume of tumors in treated mice (c) and control mice (d). In (a) and (b), the heights of the bars denote mean values of tested variables and whiskers, the SEM. As shown in (a), there were significant differences in MVD between treated and control mice, confirming pathologic response to bevacizumab in treated mice. However, final tumor volumes of (b) and Δ_vol_ of treated and untreated mice (c and d, respectively) were not significantly different. MVD, microvessel density.

Treated tumors showed a lower number of CD34 positive vessels per unit area than control tumors, that is, 2.91×10^−6^ versus 6.57×10^−6^ (*P*=0.032) confirming pharmacologically effective administration of bevacizumab. Measured directly with calipers, treated and control mice had mean Vol_cal_ of 312±72.93 mm^3^ and 352±47.5 mm^3^, respectively (*P*=0.7). There were no significant growth delays from bevacizumab treatment of treated mice, with both groups showing similar mean Δ_vol_ of 424.53±238.7% (treated) versus 434.8±117.9% (control, *P*=0.9; Fig. 1).

### Conventional imaging metrics response

Standard methods of response assessment with ^18^F-FDG PET showed no differences between treated and control mice: treated and control mice showed similar median Vol_met_ of 375±96.9 versus 384.9±60.2 mm^3^ (*P*=0.28), Met_max_ of 43.14±15.3 versus 47.6±25.7%ID/g (*P*=0.7), and Met_mean_ of 14.22±5.32 versus 17.85±7.5%ID/g (*P*=0.32; Fig. 1).

### ^18^F-FDG PET-derived texture parameter response

**Fig. 2:**
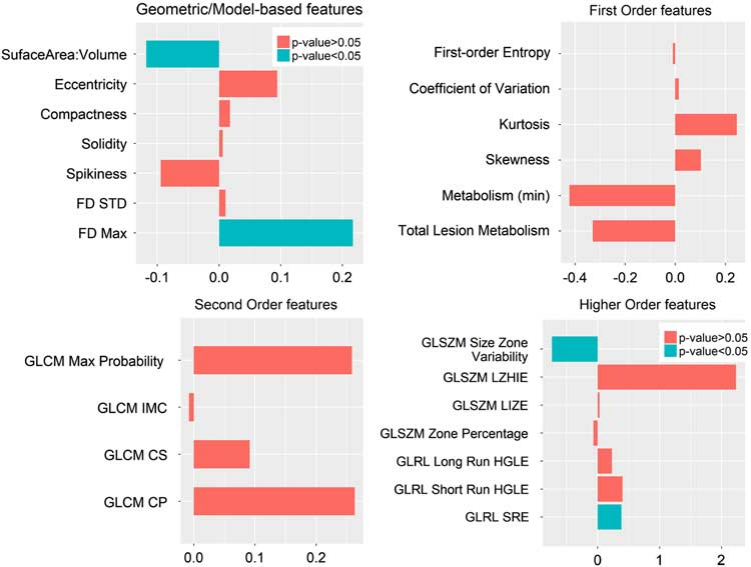
Bar plots illustrating differences in median values of individual texture parameters left after excluding highly correlated features. Four subplots are generated after grouping texture features together. Bars pointing toward the left indicate that corresponding texture features were lower in the treatment group versus the control group. Statistically, significant differences are indicated in blue. FD, fractal dimension; FD-STD, FD-standard deviation; GLCM, gray-level co-occurrence matrix; GLCM CP, GLCM cluster prominence; GLCM CS, GLCM cluster shade; GLCM IMC, GLCM information measure correlation; GLRL, gray-level run-length; GLRL long run HGLE, GLRL long run high gray-level run emphasis; GLRL-SRE, GLRL short-run emphasis; GLSZM, gray-level size zone matrix; GLSZM LIZE, GLSZM low-intensity zone emphasis; GLSZM LZHIE, GLSZM long zone high-intensity emphasis.

After excluding highly correlated texture parameters, 24 texture features remained. These were six first-order, four second-order, seven high-order statistical features, five geometric, and two model-based features (Table 1). Four variables were statistically significantly different between the two groups, that is, surface area to volume ratio (*P*=0.004), fractal dimension maximum (FD_max_; *P*=0.03), gray-level run-length short-run emphasis (GLRL-SRE; *P*=0.02), and gray-level size-zone matrix size zone variability (GLSZM-SZV; *P*=0.001). After applying the Bonferroni-adjusted *P* value cutoff (*P*<0.002), one variable remained (GLSZM-SZV; *P*=0.001). Treated mice showed GLSZM-SZV that was on average −0.74-fold (or 26%) that of control mice. Figure 2 provides a graphical summary of fold change (with respect to control group) in each of the 24 texture variables.

**Table 1:**
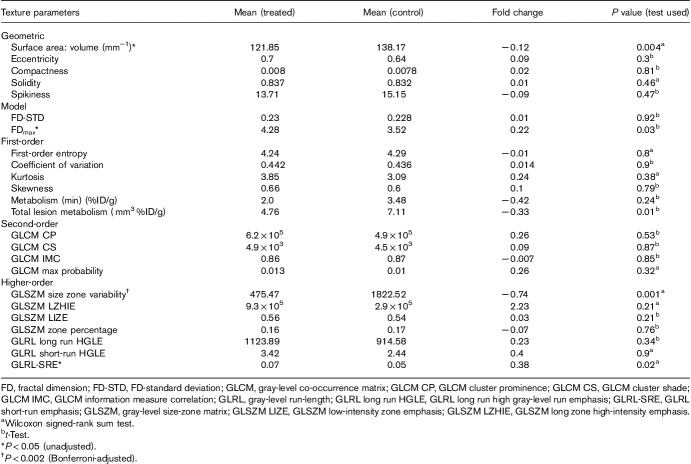
Summary differences in mean texture parameters between treated and control mice

## Discussion

In our study, we assessed conventional response biomarkers and a number of texture parameters in a mouse xenograft colorectal cancer model treated with antiangiogenic targeted agent bevacizumab. Our study was based on the premise that tumors treated with bevacizumab do not undergo changes in size early because of the cytostatic rather than cytoreductive effect of bevacizumab; however, we hypothesized that there may be changes within the tumor microenvironment secondary to the vascular remodeling following bevacizumab treatment that may be detectable and quantifiable by ^18^F-FDG PET texture analysis. We found that after 2 weeks of bevacizumab treatment, four ^18^F-FDG PET-derived texture parameters, that is, surface-to-volume ratio, FD_max_, GLSZM-SZV, and GLRL-SRE, were significantly different between treated and control mice. After applying Bonferroni correction for multiple comparisons, a single parameter, GLSZM-SZV (−0.74-fold difference; *P*=0.001), remained significant. In contrast, conventional descriptors of tumor response, that is, morphologic tumor volumes and metabolism (Met_max_ and Met_mean_), were not significantly different between the two groups of mice.

The GLSZM provides an estimation of a bivariate conditional probability density function of image distribution values 25. The more homogeneous the texture, the wider and flatter the matrix. High GLSZM-SZV is an indicator of inhomogeneity; for any given gray-level intensity, there are numerous patches of different sizes. In our study, treated mice had much smaller values of GLSZM-SZV compared with control mice, which can be interpreted as their ^18^F-FDG PET images showing little variation in sizes of isometabolic patches. Although, GLSZM-SZV by itself does not allow interpretation of the sizes of patches, the patch sizes can be inferred to be generally smaller in treated mice, based on their generally higher GLRL-SRE (0.38-fold difference; *P*=0.02) and FD_max_ (0.22-fold difference; *P*=0.03), even though both variables were excluded after applying the Bonferroni-adjusted *P* value cutoff of 0.002. GLRLs compute contiguous sequences of voxels displaying similar gray levels in given directions. A high GLRL-SRE value indicates a finely textured image dominated by short runs of voxel gray levels 26. Likewise, a high FD_max_ value corresponds to a high frequency of variation in voxel gray levels, that is, a fine texture 27,28. Finally, we found a surface-to-volume ratio to be significantly lower in the treatment group (−0.12-fold difference; *P*=0.004). This finding suggests that treated tumors became more compact and approached a spheroidal, as opposed to irregular, shape. Looking at these combinations of geometric and texture variables, it appears that ^18^F-FDG activity in treated tumors was more compact and exhibited finer variation spatially, whereas untreated tumors were more irregular and exhibited a coarser metabolic texture.

There are only a few reports investigating the metabolic and functional effects of early bevacizumab treatment as monotherapy. These studies are generally in agreement with ours. Willett *et al.* 2 monitored six patients with rectal cancer on treatment with bevacizumab. After 12 days of bevacizumab treatment, the authors found that only one of five patients experienced tumor regression and another one patient experienced a decrease in tumor ^18^FDG uptake, the rest showing stable tumor sizes and metabolic activity respectively. In contrast, tumor perfusion decreased by 40–45% and blood volume by 16–39% in most patients. The authors did not test spatial metabolic texture indices as response biomarkers, however. Our findings based on caliper and ^18^F-FDG PET measurements of tumor volumes and Met_max_, as well as Metmean, are in concordance with the results reported by Willet and colleagues. Kim *et al.* 3 measured CT-derived flow parameters and ^18^F-FDG PET-derived SUV_max_, SUV_mean_, total lesion glycolysis, gray-level co-occurrence matrix (GLCM) entropy, and GLCM homogeneity in a case–control rabbit VX2 tumor model (used to model hepatocellular carcinoma) 29. Serial imaging performed up to 14 days following treatment with bevacizumab did not show any significant differences between the two groups of rabbits in any of the ^18^F-FDG PET-derived metrics, whereas CT-derived blood flow and blood volume were different. In our study, we also did not find significant differences in Met_max_, Met_mean_, or first-order entropy, although we found GLSZM-SZV to be significantly different between the two groups – not tested by Kim and colleagues. We believe that GLCM-derived features extract different textural information compared with GLSZM-derived features, as indicated by the absence of correlation between GLCM-derived and GLSZM-derived features in our study – GLCM entropy was found to be correlated with first-order entropy (*r*=0.87; *P*=0.001) and hence only first-order entropy was retained for further analysis.

A potential limitation of this study is that our sample size of 14 mice was relatively small, as is typical of xenograft studies 30, and it is possible that further texture features could have shown statistical significance with a larger cohort. Furthermore, our findings only reflect changes in tumor metabolism in response to bevacizumab, whereas, in typical clinical scenarios, bevacizumab is given in combination with chemotherapy. Nonetheless, we believe that quantification of tumor size and metabolic effects of bevacizumab monotherapy are useful to enable elucidation of its relative contribution (or lack thereof) in different scenarios of combination treatments.

## Conclusion

The findings from this exploratory study suggest that early during treatment with bevacizumab, responding tumors may undergo metabolic changes in the microenvironment manifesting as a transition from coarse to fine texture of ^18^F-FDG distribution and from an irregular to a more compact shape. In contrast, tumor sizes and maximum (or mean) metabolism do not change significantly during early bevacizumab treatment and are not reliable biomarkers of early response. These preliminary findings will need prospective evaluation in human studies, but offer novel biomarkers of treatment response to bevacizumab treatment that may be more accurate than conventional size or metabolic activity parameters.
